# A Baculovirus Immediate-Early Gene, *ie1*, Promoter Drives Efficient Expression of a Transgene in Both *Drosophila melanogaster* and *Bombyx mori*

**DOI:** 10.1371/journal.pone.0049323

**Published:** 2012-11-13

**Authors:** Mika Masumoto, Takahiro Ohde, Kunihiro Shiomi, Toshinobu Yaginuma, Teruyuki Niimi

**Affiliations:** 1 Graduate School of Bioagricultural Sciences, Nagoya University, Chikusa, Nagoya, Japan; 2 Division of Biology, College of Liberal Arts and Sciences, Kitasato University, Sagamihara, Kanagawa, Japan; 3 Faculty of Textile Science and Technology, Shinshu University, Ueda, Nagano, Japan; University of Dayton, United States of America

## Abstract

Many promoters have been used to drive expression of heterologous transgenes in insects. One major obstacle in the study of non-model insects is the dearth of useful promoters for analysis of gene function. Here, we investigated whether the promoter of the immediate-early gene, *ie1*, from the *Bombyx mori* nucleopolyhedrovirus (BmNPV) could be used to drive efficient transgene expression in a wide variety of insects. We used a *piggyBac*-based vector with a 3xP3-DsRed transformation marker to generate a reporter construct; this construct was used to determine the expression patterns driven by the BmNPV *ie1* promoter; we performed a detailed investigation of the promoter in transgene expression pattern in *Drosophila melanogaster* and in *B. mori*. *Drosophila* and *Bombyx* belong to different insect orders (Diptera and Lepidoptera, respectively); however, and to our surprise, *ie1* promoter-driven expression was evident in several tissues (e.g., prothoracic gland, midgut, and tracheole) in both insects. Furthermore, in both species, the *ie1* promoter drove expression of the reporter gene from a relatively early embryonic stage, and strong ubiquitous *ie1* promoter-driven expression continued throughout the larval, pupal, and adult stages by surface observation. Therefore, we suggest that the *ie1* promoter can be used as an efficient expression driver in a diverse range of insect species.

## Introduction

Recent advances in germ-line transformation of non-drosophilid insects have opened up new opportunities for analyzing gene function. However, in non-model insects, a major obstacle to such analyses is the dearth of useful promoters for transgene expression. Reportedly, various promoters from several insect species can be used to express marker genes or foreign genes in transgenic insects (see [1]). The *piggyBac* vector is the most widely used transformation vector in insects, and many promoters have been used to drive gene expression from this vector. For example, the 3xP3 promoter [2], [3], *Bombyx mori* cytoplasmic *actin* (*BmA3*) promoter [4], [5], [6], *Gryllus bimaculatus* cytoplasmic *actin* promoter [7]. *Drosophila melanogaster heat shock protein 70* (*hsp70*) promoter [6], [8], [9], *B. mori* nucleopolyhedrovirus (BmNPV) *immediate-early gene 1* (*ie1*) promoter [10], *Autographa californica* multiple nucleopolyhedrovirus (AcMNPV) *ie1* promoter [11], [12], *Tribolium castaneum α-Tubulin1* promoter [13], *B. mori* and *Antheraea yamamai fibroin H chain* promoter [14], *B. mori sericin 1* promoter [15], *B. mori cecropin B* promoter [16], *B. mori bombyxin* and *prothoracicotropic hormone* (*PTTH*) promoter [17], and *D. melanogaster polyubiquitin* promoter [18], [19], [20] can each drive gene expression from the *piggyBac* vector. Unlike most of these promoters, which are derived from naturally occurring, constitutively expressed genes, the 3xP3 promoter was constructed artificially based on the sequence of Pax-6 binding sites; Pax-6 is an evolutionary conserved transcription factor that functions in eye development in metazoans, and the 3xP3 promoter drives expression in visual systems [21], [22]. Based on studies of transgenic animals, the eye-specific expression driven by 3xP3 promoter is highly conserved not only in diverse insect orders of Diptera, Lepidoptera and Coleoptera [1], but also in planarians [23].

While various promoters have been used to drive transgene expression in some insects, a more versatile and efficient promoter is necessary for expression of transgenes in a wider variety of insects beyond insect orders. Here, we examined the expression pattern driven by the BmNPV *ie1* promoter in fly and silkworm as a candidate for an efficient expression driver for a wide variety of insects. The *ie1* gene is present in all lepidopteran nucleopolyhedrovirus (NPV) genomes that have been sequenced; the *ie1* gene product acts as an transactivator of some early viral genes and is essential for viral DNA replication ([24], reviewed in [25], [26], [27]). The *ie1* promoter contains a TATA box and is transcribed via RNA polymerase II by host cells upon baculovirus infection; therefore, the promoter itself can function in uninfected cells that lack other virus-encoded factors [28]. The *ie1* promoter is a strong promoter that is used to express foreign genes in cell lines derived from various insect species [29], [30]. Therefore, we focused on using the *ie1* promoter to express transgenes at high levels in insects, and to this end, we examined the baseline *in vivo* expression pattern driven by the *ie1* promoter. Several viral factors (e.g., IE0, IE1, and IE2) regulate transcription from the *ie1* promoter [31], [32]; moreover, the *ie1* promoter is reportedly activated by hormones of host insects. Treatment of uninfected insect cells in culture or of fifth instar silkworm larvae with ecdysone, a juvenile hormone analogue (JHA), or both resulted in increased *ie1* promoter activity [33]. Furthermore, the ecdysone-responsive *cis*-acting elements have been identified within the BmNPV *ie1* promoter by using recombinant BmNPVs [34].

The AcMNPV *ie1* promoter fused with homologous region 5 (*hr5*) enhancer from the same virus was used to drive expression of enhanced green fluorescent protein (EGFP) in the malaria mosquito, *Anopheles gambiae* [12], and DsRed in the Mediterranean fruit fly, *Ceratitis capitata* [11]. Furthermore, the BmNPV *ie1* promoter has been used to drive expression of the *B. mori doublesex* gene [10]. However, basal transgenic (i.e., in the absence of baculovirus infection) *ie1* promoter activity is still largely uncharacterized *in vivo*. Here, we examined in detail the expression patterns driven by the BmNPV *ie1* promoter in *D*. *melanogaster* and *B*. *mori*; these species belong to different insect orders (Diptera and Lepidoptera, respectively). Surprisingly, the *ie1* promoter drove very similar expression patterns in these two insect species. For both insects, EGFP fluorescence driven by the *ie1* promoter was first present during embryogenesis; also for both insects, strong *ie1* promoter*-*driven EGFP fluorescence was evident throughout the body in each developmental stage that followed larval hatching. Thus, we suggest that the *ie1* promoter may be useful for efficient expression of transgenes in a wide variety of distantly related insect species.

## Materials and Methods

### Insects

*D. melanogaster* were reared on a standard medium at 25°C. Mutant embryos, *yellow white* (*y ac w^1118^*), were injected with DNA to generate germ-line transformants and transgenic fly strains.

A bivoltine strain (Daizo) of the silkworm, *B. mori*, was used for DNA injections and to generate germ-line transformant and transgenic strains. Silkworm larvae were reared on a diet of fresh mulberry at 25°C. The silkworm embryos were staged according to morphological markers as described by Ohtsuki [35] and Morita *et al.* [36].

### Plasmid Construction

**Figure 1:**
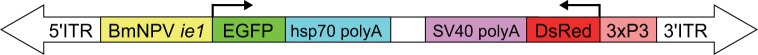
Schematic representation of the *piggyBac*-based BmNPV *ie1* promoter reporter constructs. This construct was designated pBac[BmNPV *ie1*-EGFP, 3xP3-DsRed]. A fragment containing the sequences –631 to –2 bp upstream of the codon encoding the translational start site of BmNPV *ie1* was used as the BmNPV *ie1* promoter and to drive expression of EGFP. DsRed was under the control of 3xP3 and was used as the transformation marker. Abbreviations: ITR, inverted terminal repeats of *piggyBac*; hsp70 polyA, hsp70 polyadenylation signal; SV40 polyA, SV40 polyadenylation signal.

We constructed a *piggyBac*-based vector to use in reporter assays of BmNPV *ie1* promoter activity (pBac[BmNPV *ie1*-EGFP, 3xP3-DsRed]; Fig. 1). To create restriction sites at both ends of the BmNPV *ie1* promoter (–631 ∼ –2 from the translational initiation Met codon), polymerase chain reaction (PCR) was performed using BmNPV genomic DNA as a template, a primer set comprising the RE-ie1-1 primer (5′-AAGCTTAGATCTGGCCGGCCGATTTGCAGTTCGGGACAT-3′) with *Hin*d III-*Bgl* II- *Fse* I site at 5′ end and the RE-ie1-2 primer (5′-CCATGGTCGTTTGGTTGTTCACGAT-3′) with *Nco* I site at 5′ end, and a high-fidelity DNA polymerase (Pyrobest; Takara, Japan). The resultant PCR product was subcloned into the *Eco*R V site of the pBluescript KS+ vector, and the expected nucleotide sequences were confirmed by DNA sequencing. This recombinant plasmid was then cut with *Bgl* II and *Nco* I, and the fragment containing BmNPV sequences were inserted into the *Bam*H I*-Nco* I site of the pSL[*hsp27mp-NLS-EGFP*] vector [37]. Since *Bam*H I and *Nco* I sites were contained at the both ends of the *hsp27mp-NLS* fragment, the *hsp27mp-NLS* fragment was removed from pSL[*hsp27mp-NLS-EGFP*] by cutting with *Bam*H I and *Nco* I. Thus, the BmNPV *ie1* promoter directly connected to the start codon of *EGFP*. A *Asc* I-*Fse* I fragment containing the BmNPV *ie1*-EGFP sequences was treated with T4 DNA polymerase to create blunt ends and then ligated to the *Asc* I sites (which had be filled in) of pBac[*3xP3-DsRedaf*] [1]; the resultant recombinant construct was designated pBac[BmNPV *ie1*-EGFP, 3xP3-DsRed].

**Figure 2:**
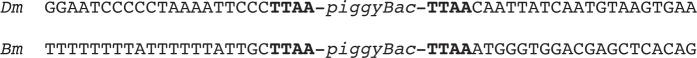
Genomic DNA sequences surrounding *piggyBac* insertions. The flanking sequences of *piggyBac* insertion in *D. melanogaster* and *B. mori* have 100% identity with the genome DNA sequences of chromosome 3L and Bm_scaf 21 in chromosome 17, respectively. Abbreviations: Dm, *Drosophila melanogaster*; Bm, *Bombyx mori*.

### Sequencing and Sequence Analysis

For each final recombinant construct, sequences derived from PCR products or from fragments cut with a restriction enzyme (i.e., any sequence that might have been altered during cloning procedures) were confirmed using the dideoxy chain-termination method by an automatic DNA sequencer (CEQ 2000XL; Beckman Coulter, USA). Sequence analysis was carried out using a DNASIS system (Hitachi Software Engineering, Japan).

### Generation of Germline Transformants

To obtain transgenic fruit flies and transgenic silkworms that carried the BmNPV *ie1* promoter construct, we injected plasmid mixtures containing 500 ng/µl of the BmNPV *ie1* promoter construct and 300 ng/µl of a helper plasmid (pshp-pBac, [18]) in injection buffer (5 mM KCl, 0.1 mM K_2_HPO_4_, pH 7.8), into the posterior pole of *Drosophila* embryos or into the posterior-ventral midline of *Bombyx* embryos during the syncytium stage of development (2 h and 12 h after oviposition at 25°C, respectively). Microinjections were performed under a dissection microscope (Stemi 2000, Carl Zeiss, Germany) using a micromanipulator (Narishige, Japan) and FemtoJet (Eppendorf, Germany), and injections into *Bombyx* embryos were performed with a special glass needle (uMPm-02, Daiwa Union, Japan) [38]. Transgenic flies were generated as described by Rubin and Spradling [39]. To identify *Drosophila* transformants, G_1_ adults were screened for expression of the 3xP3-driven DsRed transformation marker in the eyes. To identify *B*. *mori* transformants, late stage G_1_ embryos were screened for DsRed expression in the central nervous system (CNS). After G_2_ generations, we screened EGFP positive embryos at each generation to maintain the transgenic *B. mori* lines. All *B. mori* transgenic lines showed similar patterns and intensities of EGFP expression under the control of the *ie1* promoter. The transgenic *D. melanogaster* lines and the transgenic *B. mori* lines have stably transmitted the transgene for more than 80 and 10 generations, respectively.

**Figure 3:**
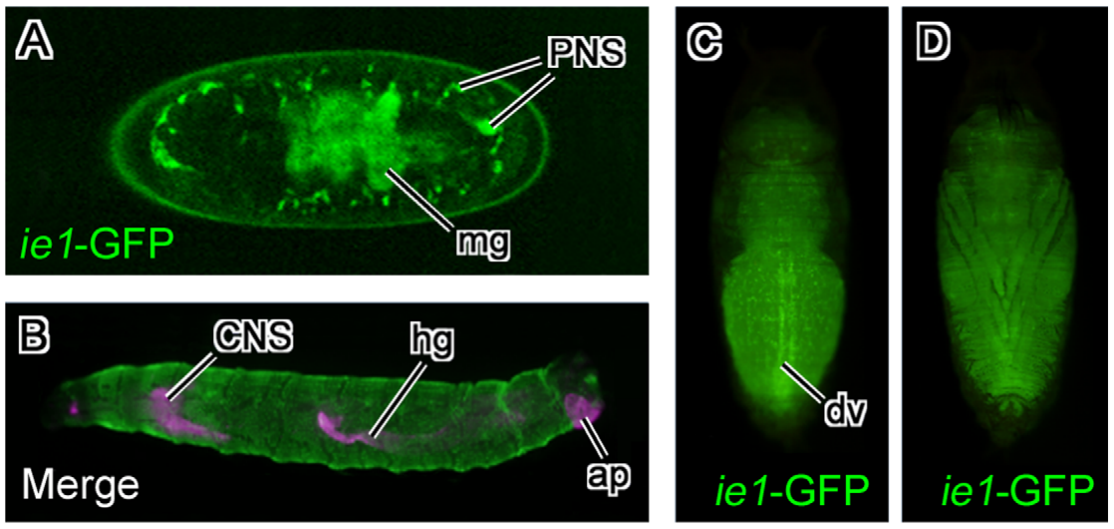
Expression pattern of the BmNPV *ie1*-EGFP transgene in fruit flies. (A) Embryos at stage 16. EGFP fluorescence was primarily evident in midgut and peripheral nervous system. (B) Merged image of BmNPV *ie1* promoter-driven EGFP expression and 3xP3-driven DsRed expression in a fruit fly larva. Strong EGFP expression was evident throughout the whole body. (C, D) BmNPV *ie1* promoter-driven EGFP expression in a fruit fly pupa. EGFP expression was evident throughout the whole body, and strong punctate expression was evident inside the body. (C) Dorsal view. Intense EGFP fluorescence was evident along the dorsal vessel. (D) Ventral view. In (A) and (B), anterior is to the left. In (C) and (D), anterior is up. Abbreviations: ap, anal plate; CNS, central nervous system; dv, dorsal vessel; hg, hindgut; mg, midgut; PNS, peripheral nervous system.

**Figure 4:**
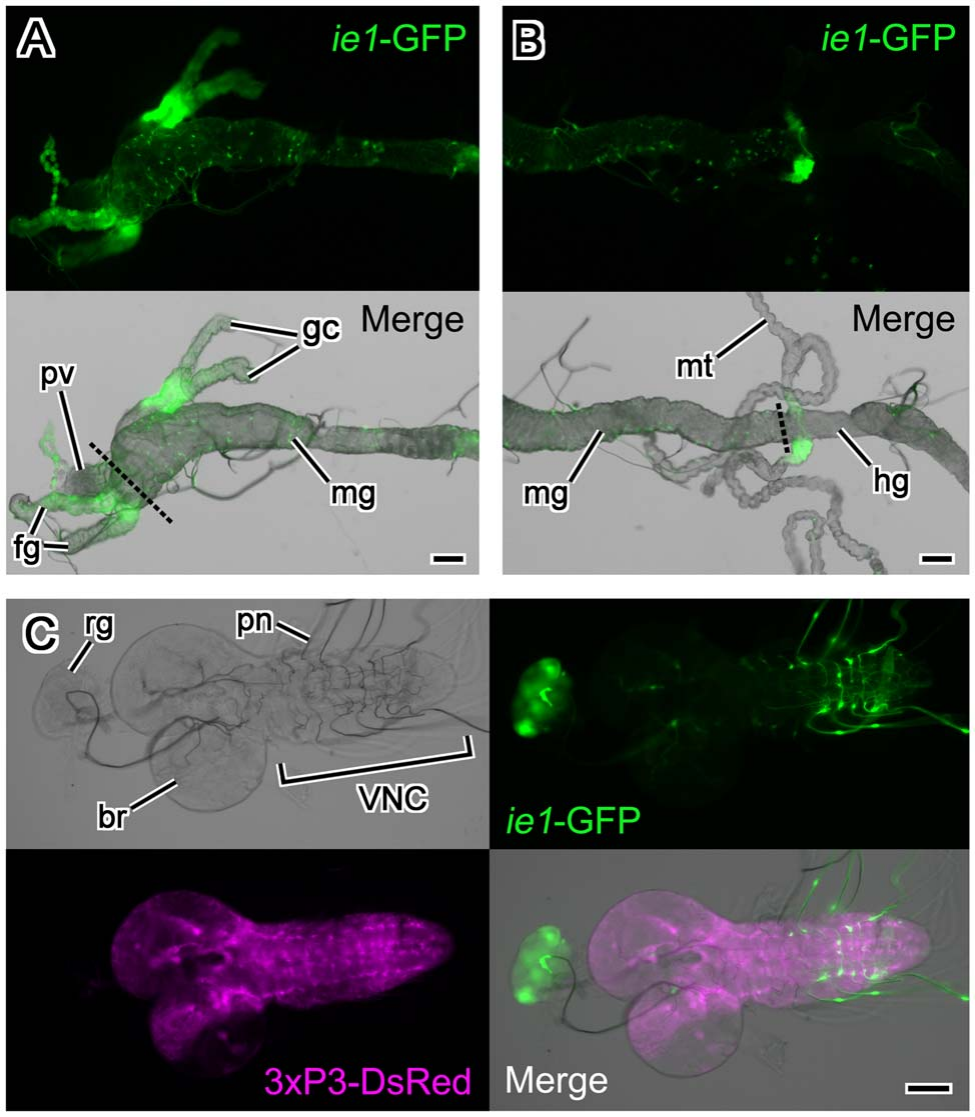
Expression pattern of the BmNPV *ie1*-EGFP transgene in tissues of a 3rd instar fly larva. (A) Foregut and midgut. EGFP expression was evident in the foregut and the gastric caecum. The dashed line indicates the boundary between the foregut and midgut. (B) Midgut and hindgut. EGFP expression was evident in the section of the Malpighian tubule that was attached to hindgut. The dashed line indicates the boundary between the midgut and hindgut. (A, B) Upper panels show EGFP fluorescence while lower panels show merged images of the transmitted light and EGFP fluorescent. EGFP was also detected along tracheoles that attached to the midgut. (C) Central nervous system and ring gland. EGFP expression was strong in the prothoracic gland region of the larval ring gland. EGFP signals were detected in the peripheral nerves emanating from the abdominal neuromere. DsRed driven by the 3xP3 promoter was expressed in the ventral nerve cord and throughout the brain except within the optic lobe. The lower right panel shows the merged images of transmitted light, EGFP fluorescence, and DsRed fluorescence. In all panels, anterior is to the left. Abbreviations: br, brain; fg, foregut; gc, gastric caecum; hg, hindgut; mg, midgut; mt, Malpighian tubule; pn, peripheral nerves; pv, proventriculus; rg, ring gland; VNC, ventral nerve cord. Scale bars = 100 µm.

### Inverse PCR

Genomic DNA was extracted from adult flies or diapausing silkworm eggs using the QIAamp Tissue Kit (Qiagen, Germany). The following procedures for inverse PCR were performed as described in a previous study [40].

### Detection of EGFP and DsRed Fluorescence

*Bombyx* embryos (G_2_, G_4_ and G_9_ generation) were dissected from their eggs in 0.75% NaCl, fixed with a chilled PLP fixative (4% paraformaldehyde, 30 mM NaPO_4_, 10 mM NaIO_4_, and 75 mM lysine, pH 6.8), and observed in 0.75% NaCl. *Bombyx* larvae (G_2_, G_3_, G_4_, G_5_, G_7_ and G_10_ generation) were dissected and observed in 0.75% NaCl. *Drosophila* embryos and larvae (G_4_ and several later generations) were dissected in PBS (137 mM NaCl, 2.68 mM KCl, 10.14 mM Na_2_HPO_4_, and 1.76 mM KH_2_PO_4_) and then fixed with 4% paraformaldehyde in PBS; dissected specimens were observed in 60% glycerol in PBS. EGFP and DsRed fluorescences were observed with a fluorescent stereomicroscope (MZ FLIII; Leica, Germany) or a fluorescent microscope (BZ-9000; KEYENCE, Japan).

**Figure 5:**
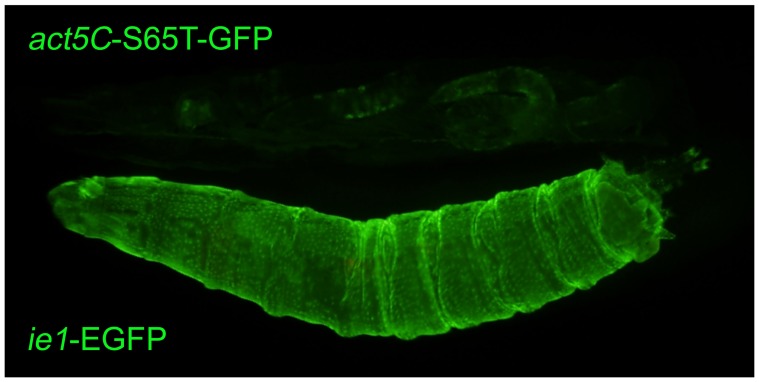
Comparison of the expressions driven by the *D. melanogaster actin5C* and the BmNPV *ie1* promoter. The *D. melanogaster actin5C* promoter drove expression of S65T-GFP (upper larva), while the BmNPV *ie1* promoter drove expression of EGFP (lower larva). These two larvae were examined side by side within the same field of view. Anterior is to the left.

**Figure 6:**
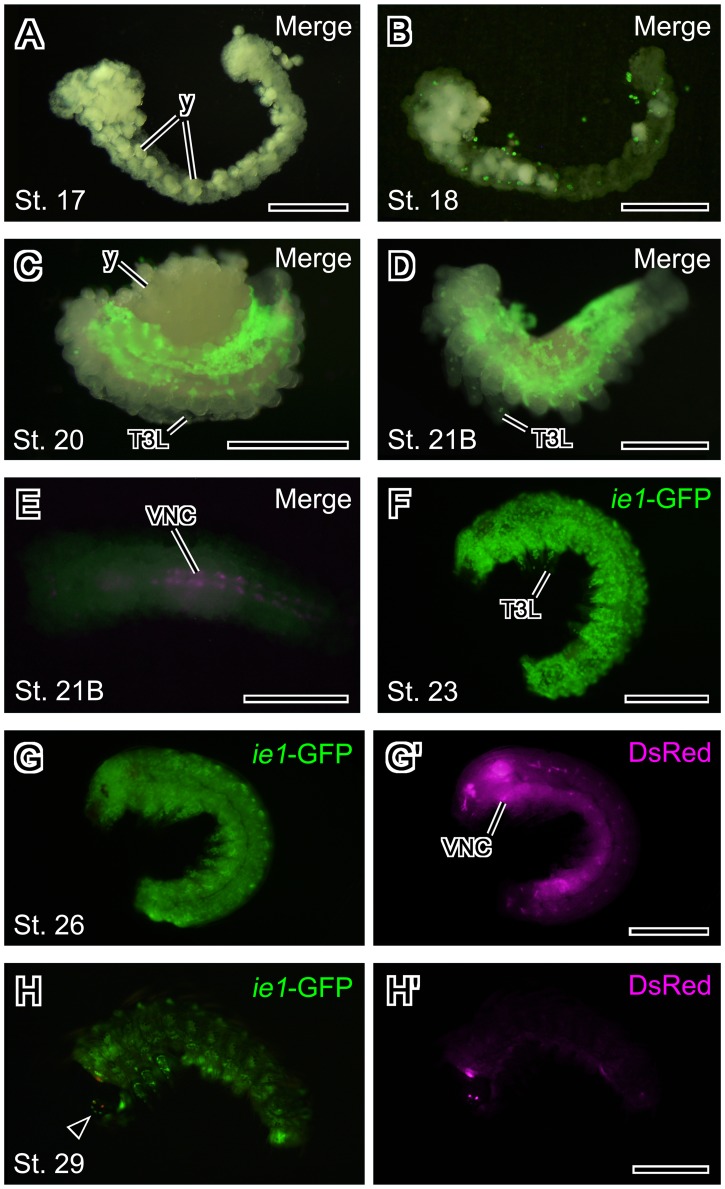
Expression pattern of the BmNPV *ie1*-EGFP transgene in silkworm embryos. (A–D) Merged images of EGFP fluorescence and epi-illumination. (A) Stage 17 embryo. EGFP expression driven by the *ie1* promoter was not evident in stage 17 embryos. Most yolk had been removed. (B) Stage 18 embryo. EGFP expression was evident in some yolk cells at this stage. (C) Stage 20 embryo (dorsal closure has not complete). EGFP expression was detected in the lateral and dorsal region. (D) Stage 21B embryo. EGFP fluorescence extended to ventral region. Most yolk had been removed. (E) Ventral view of a stage 21B embryo. Merged images of DsRed fluorescence and epi-illumination. The DsRed transformation marker was evident in the developing ventral nerve cord from this stage. (F) Stage 23 embryo (dorsal closure has completed). EGFP expression was detected throughout the whole embryo at this stage. (G, G’) Stage 26 embryo. (H, H’) Stage 29 embryo. EGFP and DsRed fluorescences were obscured by the pigmentation in the cuticle. In all panels, anterior is to the left. All panels except the panel E present a lateral view. Abbreviations: T3L, the 3rd thoracic leg; VNC, ventral nerve cord; y, yolk. Arrowhead indicates stemma. Scale bars = 500 µm.

## Results

### Construction and Transformation of the BmNPV *ie1* Reporter Construct Using the *piggyBac* Vector

To characterize the expression profile of the BmNPV *ie1* promoter in transgenic insects, we constructed a *piggyBac*-based vector (pBac[BmNPV *ie1*-EGFP, 3xP3-DsRed]; Fig. 1) to use in reporter assays. A BmNPV *ie1* promoter fragment containing sequences from –631 to –2 upstream of the codon encoding the *ie1* translational start site was used to drive expression of EGFP. This promoter region from the BmNPV *ie1* gene contained promoter regions that have been well characterized [33], [34] and showed 96.2% identity to the promoter region from the AcMNPV *ie1* gene [41], which has been used for expression of foreign genes [11], [12]. We used DsRed driven by the 3xP3 element as a transformation marker. The 3xP3 element drives transgene expression in *Drosophila* ocelli, ommatidia, Bolwig organs, and CNS [2], [37], [42]; this element also drives expression in the *Bombyx* stemmata, CNS, and peripheral nervous system (PNS) beginning on the fifth day of embryonic development [3].

**Figure 7:**
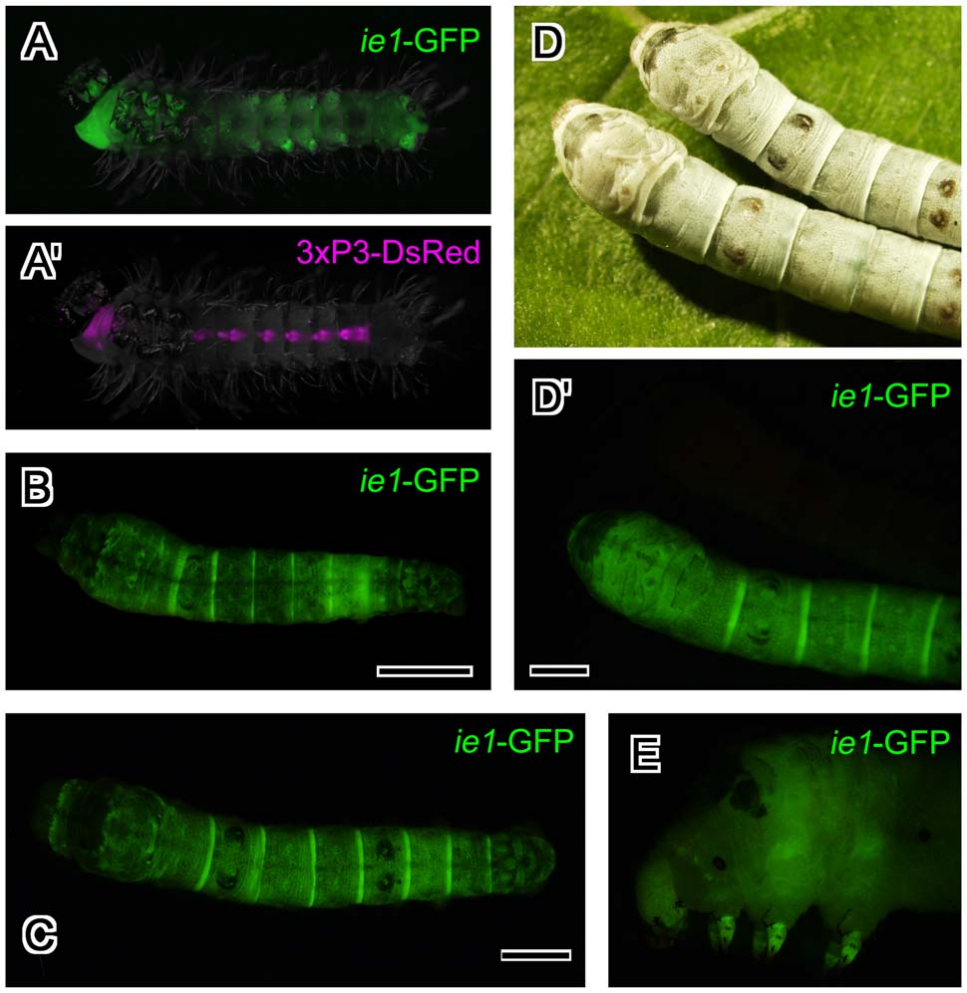
Expression pattern of the BmNPV *ie1*-EGFP transgene in silkworm larva at different stages. (A, A’) First instar larva just after hatching. BmNPV *ie1* promoter-driven EGFP expression did not overlap with 3xP3-driven DsRed expression in the ventral nerve cord. Ventral view. (B) Late 2nd instar larva. EGFP was expressed throughout the whole body and throughout all larval stages. Dorsal view. (C) Early 3rd instar larva. Dorsal view. (D, D’) 4th instar larvae. Dorsal view. Upper larva is a non-transgenic larva. (E) Head and thorax of 5th instar larva. Lateral view. Scale bars = 2 mm.

We generated transgenic flies and transgenic silkworms with this *piggyBac*-based construct. In *D. melanogaster*, of 401 injected embryos, 153 larvae hatched into 74 eclosed adults, 50 of which were fertile. These adults produced G_1_ progenies when individually crossed with *yw* adults. One G_0_ cross gave rise to several DsRed exressing progenies, resulting in a transformation efficiency of 2.0%. One line is homozygous viable and used for subsequent EGFP expression analysis.

In *B*. *mori*, of 275 injected embryos, 23 larvae hatched into 14 eclosed fertile adults. These G_0_ adults were intercrossed and their progenies were subjected to screen transformants. One G_0_ adult gave DsRed positive progenies, resulting in a transformation efficiency of 7.1%. At least 3 lines were recovered from G_1_ progenies because of the multiple insertions occurred at the transgenic G_0_. These multiple insertions of a *piggyBac* vector are frequently occurred in *B*. *mori* [4]. As we did not detect any difference in DsRed expression pattern except the intensity of DsRed expression, we did not detect any difference in EGFP expression patterns among the transgenic lines. Therefore, we maintain one transgenic line for further analysis.

We examined the genomic DNA sequences surrounding the *piggyBac* insertion site of *D. melanogaster* and *B. mori* by inverse PCR. As shown in Fig. 2, the TTAA target site of *piggyBac* was found at both ends of the *piggyBac* insertion site for all individuals, and the characteristic duplication of TTAA, which occurs following *piggyBac*-mediated transposition, was also observed. This result indicates that *piggyBac* had precisely integrated into the *D. melanogaster* and *B. mori* genome.

**Figure 8:**
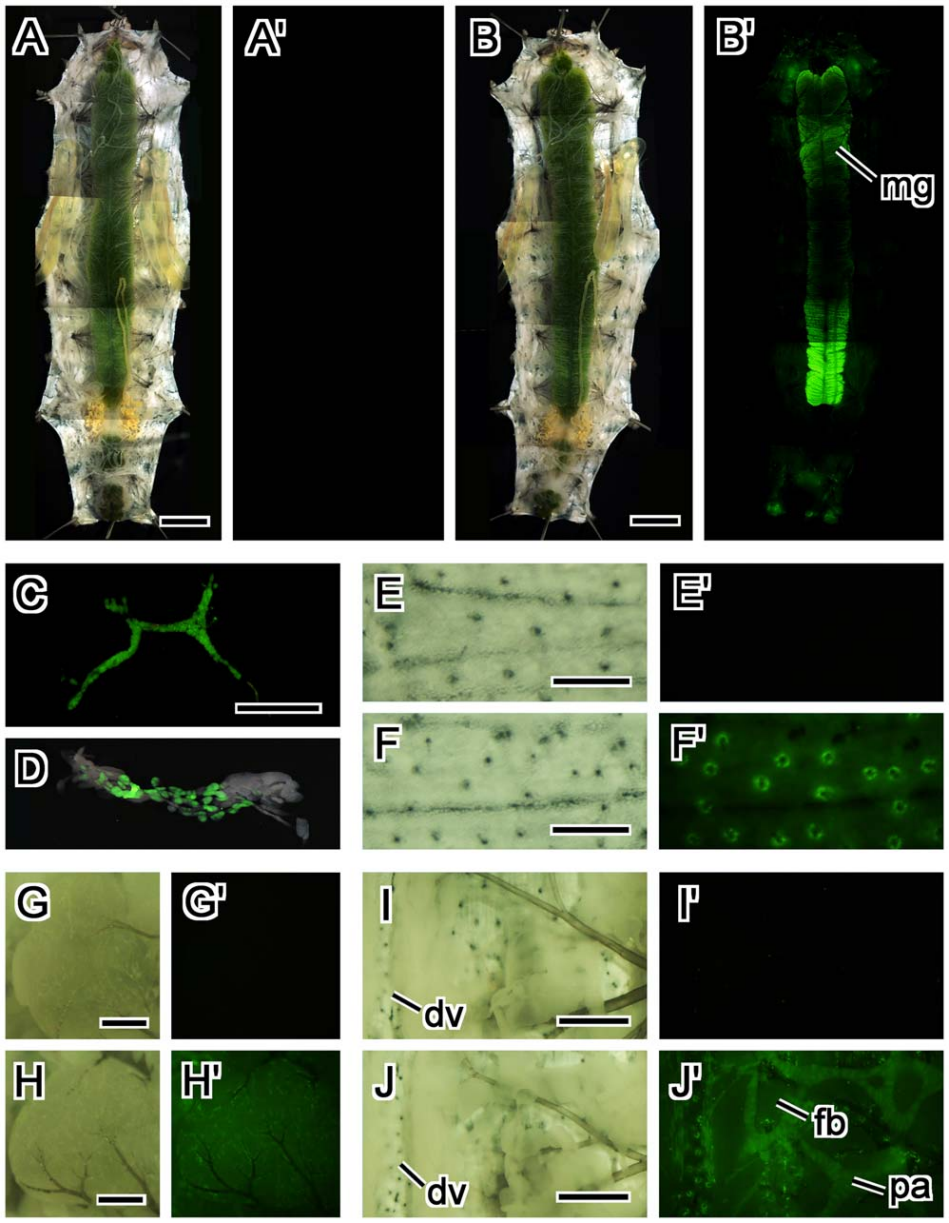
Expression pattern of the BmNPV *ie1*-EGFP transgene in tissues dissected from 5th instar silkworm larvae. (A, A’) A dissected non-transgenic 5th instar larva. (B, B’) A dissected transgenic 5th instar larva. Intense EGFP fluorescence was evident in the anterior and posterior midgut. (C–J’) A dissected 5th instar larva. (C) Prothoracic gland. (D) Merged image of the transmitted light and EGFP fluorescence in the suboesophageal body. (E–F’) Trichogen (or trichogen and tormogen) cells in the epidermis in a non-transgenic larva (E, E’) and a transgenic larva (F, F’). (G–H’) Ovary of a non-transgenic larva (G, G’) and a transgenic larva (H, H’). EGFP was evident in tracheolar cells that were attached to the ovary. (I–J’) Tissues surrounding dorsal vessel of a non-transgenic larva (I, I’) and a transgenic larva (J, J’). EGFP was evident in pericardial cells along dorsal vessel and on the alary muscle, and peritracheal athrocytes, but not in fatbody. (A, B, E–J) White light, (A’, B’, C, E’-J’) EGFP-excitation wavelength light. The images for the comparisons of non-transgenic and transgenic larvae and tissues were obtained exactly by the same conditions. Abbreviations: dv, dorsal vessel; fb, fatbody; mg, midgut; pa, peritracheal athrocytes. Scale bars = 5 mm in (A) and (B), 500 µm in (C, E, F, G, H), 1 mm in (I, J).

**Figure 9:**
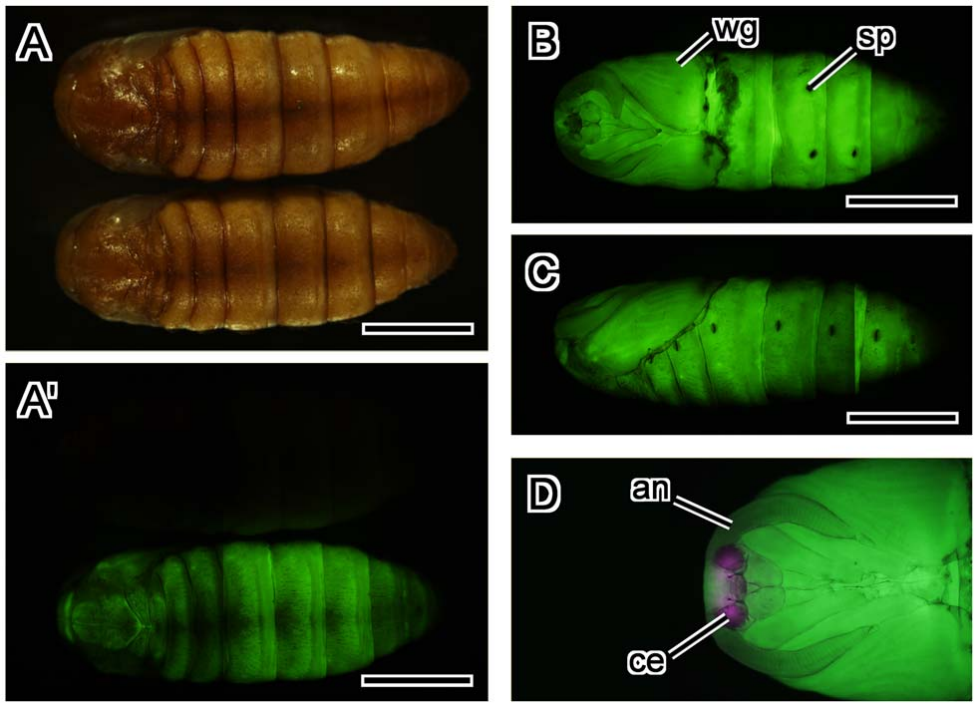
Expression pattern of the BmNPV *ie1*-EGFP transgene in silkworm pupa. (A–C) Two-day old pupa. EGFP expression was evident throughout the pupal body. (A, A’) Dorsal views under white light (A) and EGFP-excitation wavelength light (A’). Upper pupa is non-transgenic pupa. (B) Ventral view. (C) Lateral view. (D) A ventral view of the head and thorax of 4-day old pupa. DsRed expression was evident in the compound eyes, whereas EGFP was not. Abbreviations: an, antenna; ce, compound eye; sp, spiracle; wg, wing. Scale bar = 5 mm.

### EGFP Expression Driven by the BmNPV *ie1* Promoter in Transgenic Flies

We examined EGFP expressed from the BmNPV *ie1* promoter in transgenic flies by surface observation (Fig. 3). EGFP expression was detected during late embryogenesis. In stage 16 embryos (13–16 h after fertilization), EGFP fluorescence was mainly detected in midgut and the PNS (Fig. 3A). In wandering 3rd instar larvae, strong EGFP expression was evident throughout the body (Fig. 3B). In contrast, DsRed expression was observed in the Bolwig organ, CNS, hindgut, and anal plate as previously described (Fig. 3B; [37], [42]). In pupa, EGFP expression was evident throughout the whole body; punctate, strong expression was also evident in some cells within the body, and the expression along the dorsal vessel was stronger than the expression in other tissues (Fig. 3C, D).

To identify more precisely the tissue that expressed EGFP, we dissected 3rd instar larvae and observed the EGFP fluorescence using a fluorescent microscope (Fig. 4). EGFP expression was evident in the foregut, gastric caecum, and a section of Malpighian tubule (Fig. 4A, B). Also EGFP expression was evident along tracheoles that attached to the midgut, and in the peripheral nerves within the abdominal neuromere (Fig. 4A, B, C). Interestingly, EGFP expression was strong in the prothoracic gland region within the larval ring gland (Fig. 4C). DsRed was expressed in the ventral nerve cord and throughout the brain except for the optic lobe (Fig. 4C).

We also compared the expression pattern driven by the *ie1* promoter with that of a widely used, constitutive *D. melanogaster* promoter derived from the *act5C* gene. This *act5C* promoter was used to drive expression of S65T-GFP and is widely used as a GFP marker on a balancer chromosome [43]. A precise comparison of the strength of the *ie1* promoter with that of the *act5C* promoter as it is in TM3-pAct-GFP flies was difficult because the two GFP variants fluoresce with different intensities and because the transgenes were inserted at different genomic locations; nevertheless, this comparison could provide a clue to the utility of *ie1* promoter because the TM3-pAct-GFP flies are so widely used [43]. EGFP fluorescence driven by the BmNPV *ie1* promoter was much stronger than S65T-GFP fluorescence driven by the *D*. *melanogaster act5C* promoter in *D*. *melanogaster* third instar larva (Fig. 5).

EGFP fluorescence was also observed in adult flies and strong expression was detected in abdomen (data not shown).

### EGFP Expression Driven by the BmNPV *ie1* Promoter in Transgenic Silkworms

In stage 17 embryos (appendage development in the gnathal and thoracic segments), no EGFP expression was detected (Fig. 6A); EGFP expression was first detected in some yolk cells beginning in stage 18 (Fig. 6B). By stage 20 (gradual gathering of gnathal appendages), EGFP expression was detected in the lateral and dorsal regions, although the dorsal region was not completely formed in these stage 20 embryos (Fig. 6C). In stage 21B (middle blastokinesis), additional fluorescence was evident in the thoracic legs, and this fluorescence extended from the dorsal to the ventral region (Fig. 6D). Additionally, expression of DsRed, the transformation marker, was evident in the developing ventral nerve cord of stage 21B embryos (Fig. 6E). In stage 23–26 embryos, EGFP expression was evident throughout the whole embryo (Fig. 6F, G), and DsRed was detected only in the stemma and CNS (Fig. 6G’; [3]). Beginning in stage 27 embryos, EGFP and DsRed fluorescences were obscured during surface observation because the body, especially the head capsule, became pigmented (Fig. 6H, H’).

At each larval instar, strong EGFP signal was detected throughout the whole body by surface observation (Fig. 7A, B, C, D’, E). We never observed EGFP signal in wild type control larvae (Fig. 7D, D’). In first instar larva, EGFP signal was evident in stemmata, in addition to throughout the body (Fig. 7A), and DsRed signal was evident in the ventral nerve cord and stemmata (Fig. 7A’). Interestingly, EGFP signal did not overlap with DsRed signal within the ventral nerve cord.

To identify more precisely the organs that expressed EGFP, 4th and 5th instar larvae were dissected and individual organs were examined. In 4th instar larva, EGFP expression was strong in the anterior and the posterior midgut (Fig. S1B). To our surprise, EGFP expression was also strong in the pair of prothoracic glands (Fig. S1B, C). EGFP was evident in the middle and posterior part of the silk gland (Fig. S1D, E) and in the tracheole attached to the silk gland (Fig. S1F).

In 5th instar, like in 4th instar, EGFP expression was so strong in the anterior and the posterior of the midgut (Fig. 8B’) that the fluorescence was detectable event through the larval cuticle (Fig. 7E). After removing the midgut, EGFP fluorescence was detected in the pair of prothoracic glands (Fig. 8C), the suboesophageal body (Fig. 8D), and in pericardial cells along dorsal vessel and on the alary muscle (Fig. 8J’). EGFP expression was also evident in peritracheal athrocytes, but not in fatbody (Fig. 8J’). EGFP expression was not evident in either the testis (data not shown) or ovary (Fig. 8H’), but it was evident in tracheolar cells that were attached to the ovary (Fig. 8H’). Furthermore, EGFP signal was particularly strong in the trichogen (or trichogen and tormogen) cells in the epidermis (Fig. 8F’).

In pupa, BmNPV *ie1* promoter-driven expression of EGFP was evident throughout the body, especially in wings and abdomen by surface observation (Fig. 9A’, B, C). DsRed expression was evident in the compound eyes, but EGFP expression was not (Fig. 9D).

After eclosion, EGFP fluorescence was evident, but detection of fluorescence was difficult because thick scales covered the entire body (data not shown).

## Discussion

In this study, we examined the expression patterns driven by the BmNPV *ie1* promoter in *D. melanogaster* and *B. mori*. We found that, for both insects, strong EGFP fluorescence driven by the *ie1* promoter was evident throughout the whole body and throughout all developmental stages that followed larval hatching. These findings indicated that the *ie1* promoter was an efficient and strong promoter in both insect orders and, therefore, that it may be useful for heterologous gene expression or for expressing a transformation marker.

Currently, the cytoplasmic *actin* promoters are some of the most widely used constitutive promoters in transgenic insects. The *BmA3* promoter was first used as a marker of stable germ-line transformation in *B*. *mori* [4]; moreover, the *BmA3* promoter functions in the pink bollworm, *Pectinophora gossypiella* [5] and even in the sawfly, *Athalia rosae*, a Hymenoptera species [6]. Although the *BmA3* promoter successfully drives marker gene expression in these two heterologous insect species, the usefulness of this *actin* promoter may yet be species dependent. Reportedly, the promoter from the cytoplasmic *actin* (*GbA3/4*) gene of the two-spotted cricket (*G. bimaculatus*) was approximately three-fold and 80-fold more active than the promoters from the *D. melanogaster act5C* and the *D. melanogaster hsp70* genes, respectively, in a transient expression assay in *G. bimaculatus* embryos [44]. Therefore, cytoplasmic *actin* promoters in heterologous species may be less efficient than that in original species. The *BmA3* promoter has been used as a transformation marker in some insects (Lepidoptera and Hymenoptera). However, GFP driven by the *BmA3* promoter is evidently not expressed in *B. mori* embryos [4], and *BmA3* promoter-driven GFP expression does not begin until the pharate larval stage in *P. gossypiella* [5]. In contrast, EGFP fluorescence driven by the *G. bimaculatus* cytoplasmic *actin* promoter is detected from the early blastoderm stage onward in transgenic crickets [7]. Although it would be interesting to know what causes the difference between the initiation timing of the expression driven by the *BmA3* promoter and that driven by *G. bimaculatus* cytoplasmic *actin* promoter, this is another example of a species specificity of the cytoplasmic *actin* promoter. A promoter that drives gene expression late in development is not particularly useful as a marker of transformation because of the effort necessary to screen numerous hatched larvae to detect a transformant. Here, we found that, in *D. melanogaster* and in *B. mori*, the BmNPV *ie1* promoter drove strong EGFP expression from late and middle embryogenesis, respectively, throughout adulthood; therefore, we believe that this promoter may be useful for driving expression of transformation markers in a range of distantly related insect species.

Thus far, the artificial 3xP3 promoter has been the most versatile promoter for expression of transformation markers in a wide variety of insect taxa (Diptera, Lepidoptera and Coleoptera). In *B. mori*, 3xP3-driven expression is evident much earlier than is *BmA3* promoter-driven expression; 3xP3-driven expression begins on the fifth day of embryonic development at 25°C [3]. However, the BmNPV *ie1* promoter constructed for this study had advantages in the following respects. BmNPV *ie1* promoter-driven expression was evident beginning at embryonic stage 18 (3.0∼3.5 days after oviposition), which is nearly two days earlier than the first discernable 3xP3-driven expression. Moreover, expression from the *ie1* promoter was not evident in the nervous system, which is the site of 3xP3-driven expression; thus, the *ie1* promoter-driven transformation marker is compatible with 3xP3-driven transformation marker. Furthermore, the BmNPV *ie1* promoter functioned not only in *B. mori*, which is the host of BmNPV, but also in *D. melanogaster*.

Transformation markers expressed from the AcMNPV *ie1* promoter with its *hr5* enhancer were assessed in *A. gambiae* [12] and in *C. capitata* [11]. Although the expression patterns of this transformation marker were not described thoroughly for either species, expression of this marker in *A. gambiae* was evident throughout the larval thorax and abdomen and was particularly strong in the anterior stomach and salivary glands [12]; moreover, strong expression was evident throughout the larval body in *C. capitata* [11]. Here, both in *D. melanogaster* and in *B. mori*, BmNPV *ie1* promoter-driven expression was evident throughout the body of larvae and pupae, especially in the prothoracic gland, the midgut, and the tracheole. Although these two insects belong to different insect orders (Diptera and Lepidoptera) that diverged at about 240 million years ago [45], *ie1* promoter-driven expression was detected in several of the same tissues in both species (Fig. 4, 8). It is astonishing that this promoter, which is approximately 600 bp, has evolutionarily conserved tissue-specific regulatory elements. Since NPVs infect not only Lepidoptera, but also Diptera and Hymenoptera [46], the evolutionary conserved expression pattern of the BmNPV *ie1* driven EGFP marker in flies and silkworms may be concerned with something common in regard to infection by NPVs.

This *ie1* promoter may facilitate identification of *cis*-elements that function in multiple insect orders. Reportedly, the *ie1* promoter responds to ecdysone (E) and 20-hydroxyecdysone (20E) [33], [34]. Furthermore, Kojima *et al*. [34] identified an ecdysone-responsive element in the *ie1* promoter (*ie1*EcRE, 5′-GTGTTATCGACCT-3′), and this element is homologous to the ecdysone response element of *D. melanogaster* (*Dm*EcRE). The active form of ecdysteroid is 20E; ecdysteroid is first synthesized as E in the prothoracic gland or ring gland, secreted into hemolymph, and then enzymatically converted into 20E in peripheral tissues [47]. Our finding, specifically that the *ie1* promoter drove EGFP expression in prothoracic gland of *B. mori* and the ring gland of *D. melanogaster*, might be related to E synthesis. Our results might provide a clue to the identification of the transactivators that bind to the *ie1* promoter. Furthermore, the high expression of EGFP driven by the BmNPV *ie1* promoter in the prothoracic glands indicated that the transgenic silkworms with the BmNPV *ie1*-EGFP reporter can be useful for automated sorting of the cells of prothoracic glands, which are small organs and difficult to isolate by dissection.

In conclusion, the BmNPV *ie1* promoter will be useful especially in silkworms for a strong expression driver not only for functional analysis tools such as reverse genetics, tetracycline-controlled transcriptional activation system and *in vivo* RNA interference but also for a tool for future application as bioresources. Moreover, the *ie1* promoter can be used as an efficient expression driver in a diverse range of insect species and it will be useful for future basic and applied research studies.

## Supporting Information

Figure S1 **Expression pattern of the BmNPV** ***ie1*** **-EGFP transgene in tissues dissected from 4th instar silkworm larva.** (A, B) A dissected 4th instar larva. Intense EGFP fluorescence was evident in the prothoracic gland and the anterior and posterior midgut. (C) Prothoracic gland. (D, E) Silk gland. EGFP expression was evident in the middle and posterior silk gland. (E) Merged images of transmitted light and EGFP fluorescence. (F) Highly magnified image of the region within the yellow box in (D). Strong EGFP expression was evident along tracheoles attached to silk gland. Abbreviations: mg, midgut; pg, prothoracic gland. Scale bars = 5 mm in (B), 1 mm in (C) and (E), 100 µm in (F).
(TIF)
