# Supplementary figures and images for: A Baculovirus Immediate-Early Gene, ie1, Promoter Drives Efficient Expression of a Transgene in Both Drosophila melanogaster and Bombyx mori
Source: PLoS One. 2012 Nov 13;7(11):e49323. doi: 10.1371/journal.pone.0049323 (PMC3496687; doi:10.1371/journal.pone.0049323)

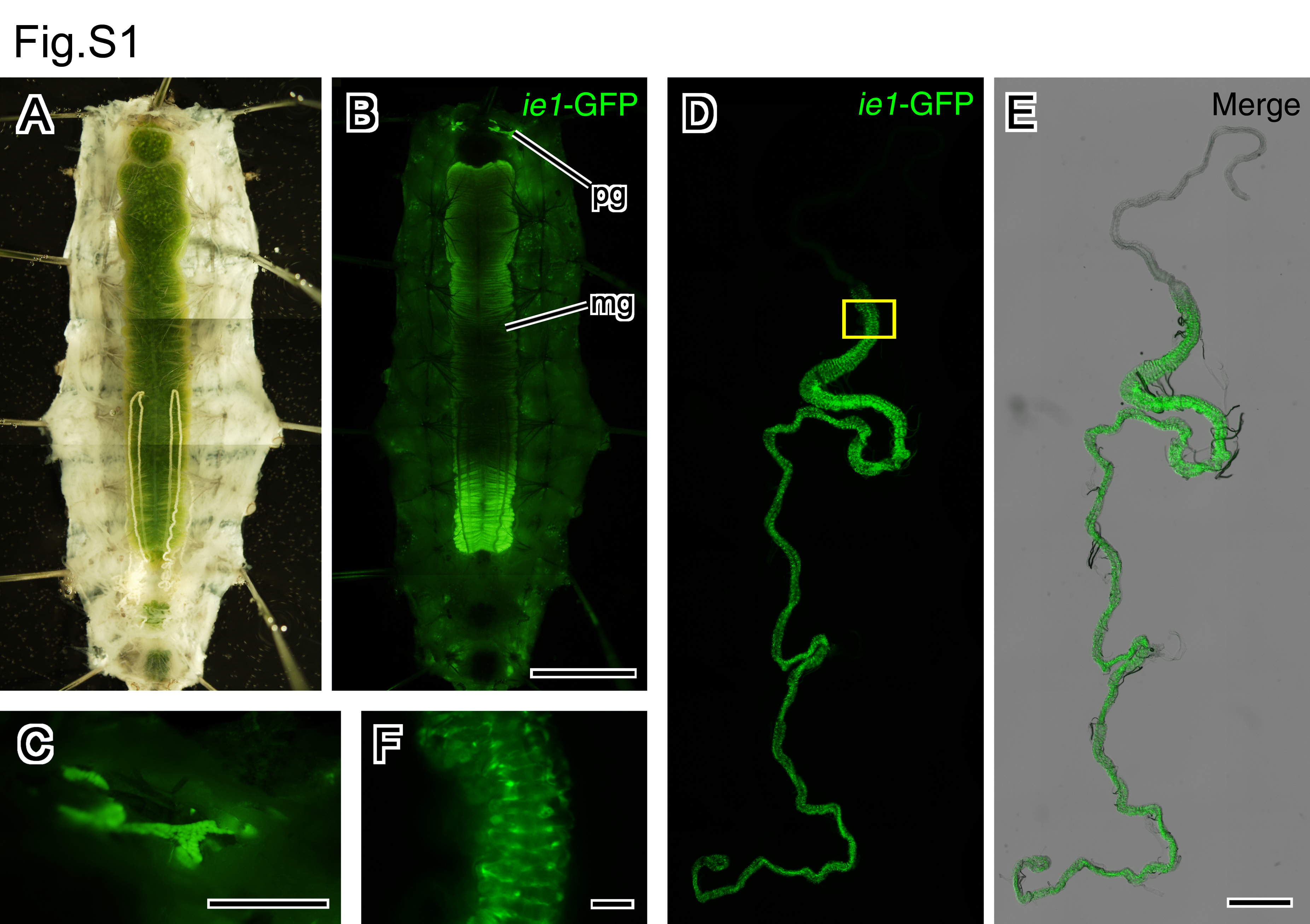

Supplement: Figure S1 — Expression pattern of the BmNPV ie1 -EGFP transgene in tissues dissected from 4th instar silkworm larva. (A, B) A dissected 4th instar larva. Intense EGFP fluorescence was evident in the prothoracic gland and the anterior and posterior midgut. (C) Prothoracic gland. (D, E) Silk gland. EGFP expression was evident in the middle and posterior silk gland. (E) Merged images of transmitted light and EGFP fluorescence. (F) Highly magnified image of the region within the yellow box in (D). Strong EGFP expression was evident along tracheoles attached to silk gland. Abbreviations: mg, midgut; pg, prothoracic gland. Scale bars = 5 mm in (B), 1 mm in (C) and (E), 100 µm in (F). (TIF) [file pone.0049323.s001.tif]
